# Combined Effects of Set Retarders and Polymer Powder on the Properties of Calcium Sulfoaluminate Blended Cement Systems

**DOI:** 10.3390/ma11050825

**Published:** 2018-05-17

**Authors:** Seongwoo Gwon, Seung Yup Jang, Myoungsu Shin

**Affiliations:** 1 School of Urban and Environmental Engineering, Ulsan National Institute of Science and Technology (UNIST), 50 UNIST-gil, Ulsan 44919, Korea; ksw4430@unist.ac.kr; 2 Department of Transportation System Engineering, Graduate School of Transportation, Korea National University of Transportation, Uiwang 16106, Korea; syjang@ut.ac.kr

**Keywords:** calcium sulfoaluminate (CSA) cement, set retarder, hydration, porosity, strength development, microstructure

## Abstract

This study investigates the effects of set retarders on the properties of polymer-modified calcium sulfoaluminate (CSA) and Portland cement blend systems at early and long-term ages. The fast setting of the cement blend systems is typically adjusted by using retarders to ensure an adequate workability. However, how the addition of retarders influences the age-dependent characteristics of the cement blend systems was rarely investigated. This study particularly examines the effects of retarders on the microstructure and strength development of polymer-modified CSA and Portland cement blend pastes and mortars from 2 h to 90 days. The macro- and microstructural properties are characterized by compression testing, powder X-ray diffraction, mercury intrusion porosimetry, and scanning electron microscopy with energy dispersive spectroscopy. The test results reveal that the use of retarders delayed the strength development of the cement blend systems at the very early age by hindering the production of ettringite, which was cumulative to the delaying effect of polymer, but it increased the ultimate strength by creating denser and finer pore structures with the evolution of hydration products.

## 1. Introduction

Calcium sulfoaluminate (CSA, Ye’elimite) cement is a promising binder alternative to Portland cement. It can be manufactured from calcium sulfate, limestone, and bauxite at a kiln temperature of about 1250 °C, which is about 200 °C lower than that of Portland cement [1,2]. Thus, the manufacturing of CSA cement gives off less CO_2_ than Portland cement; the production of 1 ton of Portland cement generates about 800 kg of CO_2_ [3,4,5]. In respect to the composition, CSA cement is usually comprised of many mineral phases such as anhydrite, belite, calcium aluminate ferrite, and gehlenite [6]. Besides such mineral phases, the manufacturing process of CSA cement clinker can incorporate many industrial by-products such as blast furnace slag and fly ash [4,7].

Besides the aforesaid environmental advantages, one important feature of CSA cement is the rapid setting and hydration at the early age between 2 and 12 h, occurring with low shrinkage and limited self-stressing [8]. Thus, CSA cement concrete may be incorporated in many structures exposed to severe weather conditions requiring the rapid setting and strength development of concrete, such as tunnel linings, bridge decks, and airport runways.

Taking advantage of the rapid hardening quality of CSA cement systems, our research group recently developed an effective method for upgrading existing ballasted railway tracks into concrete slab tracks [9,10]. This method involves the on-site injection of fresh CSA and Portland cement blend mortar into voids between existing ballast aggregates. At the early stage of developing the method, two critical problems were identified [9,11]. One was that the durability properties such as freeze-thaw resistance were inferior due to defects in the interfacial transition zones (ITZs) between reused ballast aggregates and the mortar. The other was associated with workability such that the quick hardening permitted too short of a construction time after mixing [11], which would cause the formation of many cold joints between discrete mortar placements. The durability and workability issues were treated by adding an acrylic redispersible polymer and set retarders, respectively. The addition of the polymer effectively refined the microstructure of the ITZs so as to improve the durability properties; this is the main topic of the companion paper [11]. Moreover, it was deemed that the formation of polymer films during the hydration of cement particularly enhanced waterproofness, resistance to chloride ion penetration, freezing-thawing resistance, and chemical resistance [11,12,13,14]. The addition of the set retarders delayed the setting time, as was observed similarly with the use of the polymer in the companion paper, which showed a slower growth of viscosity over time with a higher polymer ratio from rheology tests [11]. However, the effects of the retarders on the microstructure and strength development of CSA and Portland cement blend mortars in the process of cement hydration were not clearly understood, especially in the presence of the polymer. In this regard, the study of the microstructure of cement-based materials is important, because it has a direct influence on their durability and service properties in general [15,16,17,18].

In general, set retarders are categorized into two types; one has just a set-retarding function, while the other has not only a set-retarding function but also a water-reducing function, which improves workability by reducing the water demand or plasticizing the mixture [19,20]. As with the latter type of retarders, a denser, more ordered, and finer cementitious matrix can be achieved, resulting in an increased ultimate strength compared with an un-retarded mixture having no retarders [20,21,22]. Some preceding studies examined the effects of set retarders on the macroscopic strength development of Portland cement concrete [22,23,24,25,26]. However, microstructural characterizations were scarcely attempted to figure out the mechanisms of retarders, especially for CSA and Portland cement blend concrete.

Given the aforesaid concerns, the main purpose of this study is to investigate the effects of set retarders on the microstructure and strength development of CSA and Portland cement blend pastes and mortars at early and long-term curing ages (that is, 2 h to 90 days). In particular, the combined effects of retarders and polymer were scrutinized. To achieve the goal, a series of microstructural analysis techniques were employed including elemental analysis (EA), X-ray fluorescence (XRF), powder X-ray diffraction (XRD), scanning electron microscopy (SEM) with backscattered electron image (BSE), elemental energy dispersive spectroscopy (EDS), and mercury intrusion porosimetry (MIP).

## 2. Experimental Program

### 2.1. Materials

**Figure 1:**
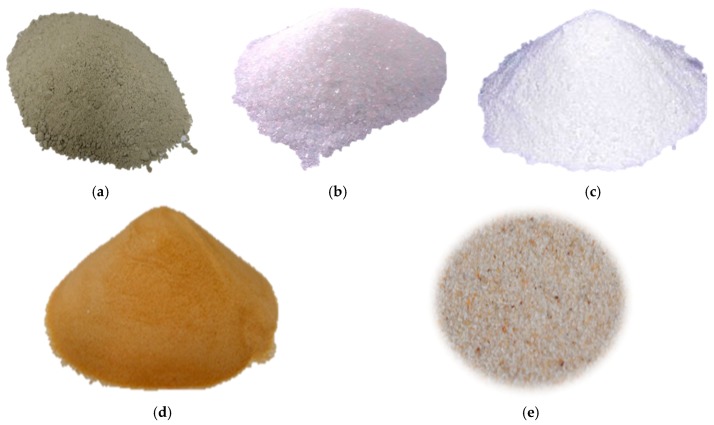
The raw materials: (**a**) cement blend; (**b**) citric acid; (**c**) zinc acetate; (**d**) redispersible polymer powder; and (**e**) quartz sand.

The cement used in this study is a premixed blend of 60 wt.% CSA-based cement containing gypsum and lime and 40 wt.% Type I Portland cement as per ASTM C150 [27] with ground granulated blast furnace slag (GGBFS). The CSA and Portland cement blend with GGBFS, set retarders, redispersible polymer powder, and quartz sand were used to produce various mixtures of cement pastes and mortars (Figure 1). Two types of set retarders were employed; citric acid and zinc acetate. The zinc acetate functions as just a set retarder, and the citric acid functions as both a set retarder and a water reducer.

For Portland cement, citric acid delays the early-age hydration by hindering the dissolutions of C_3_S and C_3_A from the cement particles [28,29,30]. For CSA cement, however, the carboxylic acid groups (–COOH) within citric acid made use of the Ca^2+^ ions from the cement to create the precipitation of Ca-complexed carboxylic acid compounds (that is, calcium citrate chain) around the surfaces of cement grains [28]. The precipitated acid compounds form hydrophobic barrier layers that hinder the very early reaction of ye’elimite [31], which causes a delayed setting and hydration [28]. In addition, the hydrophobic barrier layers improve the dispersibility of anhydrous cement grains, resulting in a finer and denser hydrated cementitious matrix [20,21,22]. In contrast, zinc acetate defers the setting and hydration by producing protective films of insoluble hydroxide around C_3_S in cement grains for Portland cement [24,32]. Thus, zinc acetate was employed separately to delay the setting and hydration of C_3_S in Portland cement, whereas the retardation of ye’elimite in CSA cement was dependent on the citric acid.

The polymer powder used as a filler was acrylic and made from condensation resin, and it consisted of 100% solid content with a bulk density of 540 kg/m^3^. The particle size distribution of the quartz sand ranged from 0.22 to 0.70 mm. Detailed characterizations for each raw material were conducted using various analysis techniques including EA, XRF, and XRD, which are presented in Section 3.1.

### 2.2. Mix Proportions

A total of ten mix proportions were tested as shown in Table 1: (1) five mixtures of mortars; and (2) five mixtures of cement pastes with no sand. The main test variables are the presence of set retarders, amount of polymer powder, and the curing age of samples. The portion of each set retarder was the same in all the mixtures excluding the two without retarders (M-0-N and B-0-N); retarder A (citric acid) and retarder B (zinc acetate) were 0.16% and 0.12% of the weight of the cement, respectively. The portion of polymer powder varied from 0 to 10% of the weight of the cement. In the mortar mixtures, the amount of the sand corresponds to 42.0% of the weight of the mixture except the polymer and retarders. As for the curing age of the samples, the tests were conducted for samples cured for 2 h to 90 days.

In the mixture label, the first letter (M or B) stands for a mortar or cement paste, the following number signifies the percentage of polymer powder, and the last letter (N or Y) stands for the absence (N) or presence (Y) of set retarders. The water-to-cement ratio was 0.38 in all the mixtures, and no plasticizer was used.

### 2.3. Sample Preparation

In preparation of the samples, at first polymer powder was dry-mixed with cement blend (or a combination of cement and quartz sand) by a mechanical stirrer. Then, the dry mixture was poured into a mixing bowl containing water in which the set retarders were dissolved in advance. The mixing continued about 10 min at a speed of 100 rpm. Finally, the plastic mixture was placed into various molds for microstructural analyses and compression tests. Then, each mold was compacted on a mechanical vibration table for a few seconds to eliminate entrained air bubbles during the mixing and placing. Excessive vibration was carefully avoided to prevent the separation of polymer powder and/or the bleeding of water.

After 24 h of curing, all hardened samples (hardened mortars and cement pastes) were demolded. Then, the curing of the hardened samples continued in an air-conditioned room with a relative humidity of 60 ± 5% and a temperature of 20–25 °C until testing. As for the preparation of the test samples for both the MIP and SEM, the hardened cylindrical sample with a 20 mm diameter and 30 mm height was cut into the pieces with a specific dimension using a low-speed diamond saw. To perform the microstructural characterizations at each curing age, further hydration was halted using acetone or isopropanol as solvents. The corresponding volumetric ratio between the sample (powder or bulk solid) and the solvent (acetone or isopropanol) was under less than 1/240. For the first three days of solvent exchange, the solvent was replaced every 24 h.

### 2.4. Test Methods

#### 2.4.1. Compression Test

Compression tests were performed on the mortar mixtures in Table 1. At least three cubic samples with sides 50 mm long were tested for each mixture. The compression tests followed ASTM C109 [33]. Additionally, various microstructural analyses were conducted to characterize the raw materials and hydrated cement pastes (HCPs) in Table 1 in both quantitative and qualitative manners.

#### 2.4.2. XRD and XRF

The XRD patterns of the raw materials and HCPs were measured to explore constituent phases and to identify crystalline phase transitions and hydration products that might occur over the curing period. The XRD tests were performed on powdered samples using a high-power X-ray diffractometer (Rigaku, Tokyo, Japan) that utilizes the emission of an incident Cu-K radiation beam (λ = 1.5418 Å) with a 2θ scanning range of 5–70° at room temperature. For all the microstructural characterizations, the powder samples were solvent-exchanged by acetone for approximately 14 days to halt further hydration and were dried in a desiccator with a constant vacuum pressure of about 4 kPa (30 mmHg) for 24 h to remove any remaining solvent [34]. The oxide compositions of the raw materials were quantified using a wavelength dispersive XRF spectrometer (Bruker S8 Tiger, Billerica, MA, USA). Regarding XRD and XRF, the powder samples were prepared using a pestle by hand. The maximum particle size is approximately 80 μm because the powdered samples were filtered through an 80 μm sieve before each test. In the XRF test, the pressed pellet samples were prepared in cylinder dies using a mechanical press without a binder.

#### 2.4.3. EA

The weight percentages of carbon, hydrogen, sulfur, and oxygen of the polymer powder were estimated using an EA (Flash 2000, Thermo Fisher Scientific, Cambridge, UK). The sample weight was approximately 1 mg. The average test result was obtained from five independent measurements.

#### 2.4.4. MIP

The pore volumes and pore size distributions of the HCPs were evaluated using a MIP (Auto Pore IV 9500, Micromeritics, Norcross, GA, USA). For each mixture, multiple cubic samples with 5 mm long sides were cut from the cores of hardened cylindrical samples for the MIP tests. The cubic samples were solvent-exchanged by isopropanol for 4 weeks to stop further hydration and subsequently dried out in the same condition as done for the XRD (Rigaku, Tokyo, Japan) samples. Preceding studies [34,35] reported that isopropanol was more effective than acetone in preserving the relatively small pores and microstructures of the solid samples. The pore size distribution was determined by measuring the amount of intruded mercury for each of the pore sizes ranging from 360 to 0.003 μm in diameter; the corresponding pressure of the mercury intrusion was increased from 0 to approximately 60,000 psi. The density, surface tension, and contact angle of mercury were set as 13.534 g/mL, 485 dynes/cm, and 130 degrees, respectively.

#### 2.4.5. SEM/EDS

The morphologies of the HCPs were examined using an ultra-high resolution field emission SEM (Hitachi S-4800, Tokyo, Japan) with EDS. For the selected HCPs in Table 1, at least three sliced samples with 3 mm thickness were cut and prepared from hardened cylindrical samples. The sliced samples were solvent-exchanged as done for the MIP samples, and were fixed in a cold-mounting with epoxy resin. The top surface of each sliced sample was ground to remove about 1-mm thickness and was polished using 6 μm, 3 μm, and 0.25 μm diamond suspensions in a row. Bulk-sectioned samples were used for all HCPs in this study. Before the SEM tests, all polished samples were coated by an osmium film to minimize charging, and to raise the contrast of obtained images.

## 3. Results and Discussion

### 3.1. Characterization of Raw Materials

**Figure 2:**
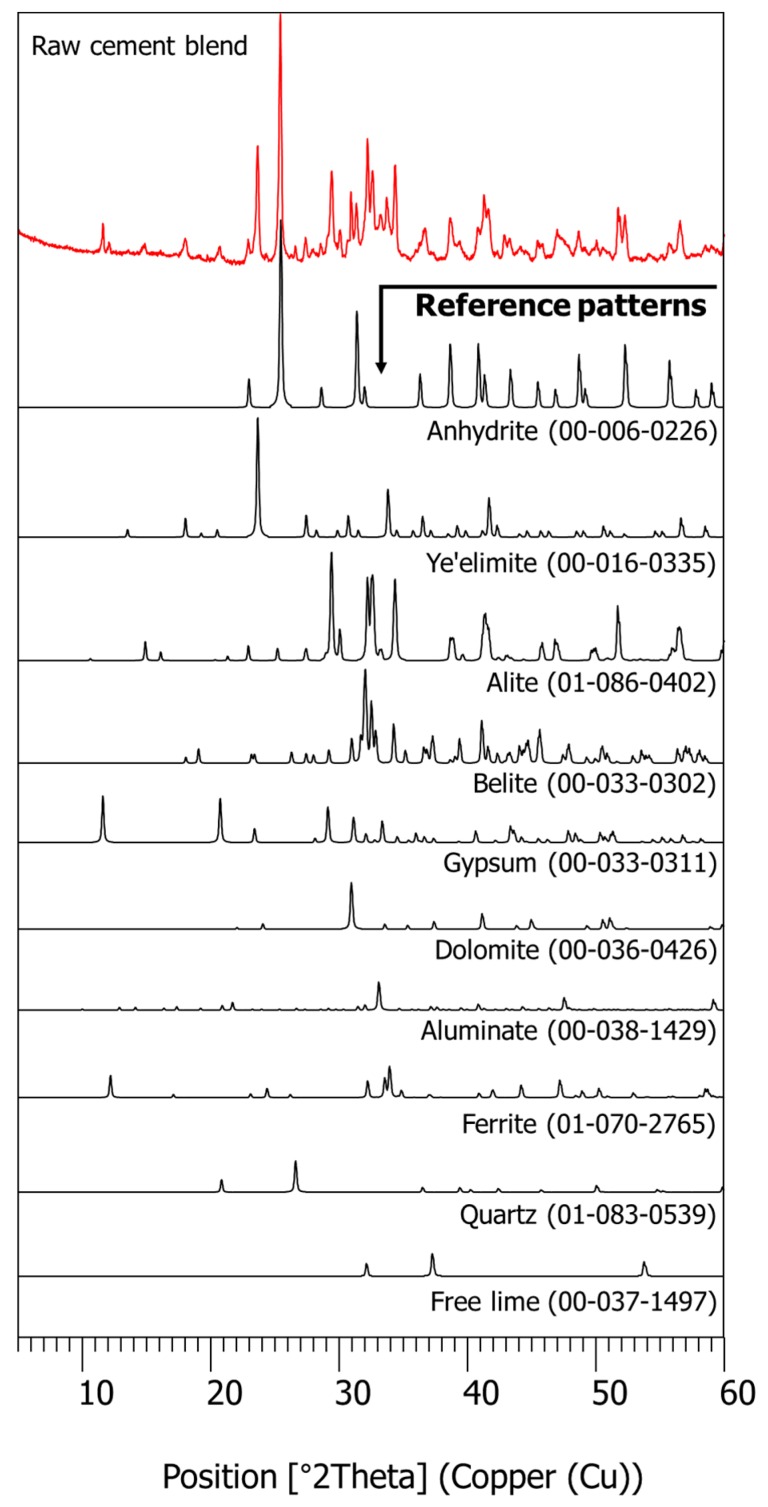
The XRD pattern of the raw cement blend.

The chemical oxide composition of the cement blend obtained from the XRF tests is given in Table 2. The cement blend contains a higher portion (that is, 11.8%) of aluminum oxide than Portland cement, which typically includes approximately 2.5–5% of aluminum oxide [36]. This accelerates the setting and hydration of the cement blend with the presence of plentiful calcium sulfate. The measured XRD pattern of the cement blend is displayed in Figure 2. The cement blend reflected a great portion of minerals. Among them, anhydrite (ICDD PDF no. 00-006-0226), ye’elimite (ICDD PDF no. 00-016-0335), alite (C_3_S) (ICDD PDF no. 01-086-0402), and belite (C_2_S) (ICDD PDF no. 00-033-0303) were found as the major phases, which is in accordance with large calcium oxide, sulfur oxide, silicon oxide, and aluminum oxide contents in the XRF (Bruker S8 Tiger, Billerica, MA, USA) results (Table 2).

In CSA cement-blended products, calcium sulfate is appended in the form of anhydrite or gypsum to adjust initial hydration reactions related to the early-age strength [37]. Preceding studies [29,38] showed that the availability of calcium sulfate ($C\bar{S}$) over ye’elimite ($C_{4}A_{3}\bar{S}$) controls the rate and quantity of hydration. However, it is often needed to slow down the setting time of CSA cement blend so that the mortar or concrete can retain a sufficient workability before placing. Thus, the role of set retarders is crucial in many applications of CSA cement blend.

**Figure 3:**
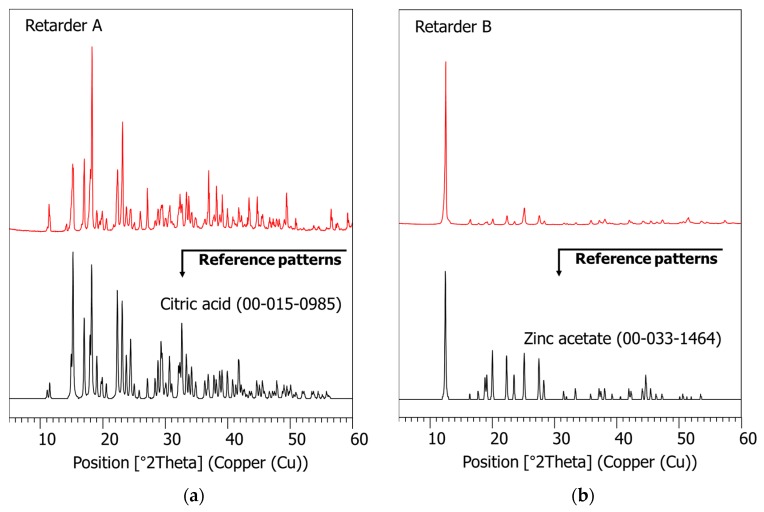
The XRD patterns of the set retarders: (**a**) citric acid; (**b**) zinc acetate.

The measured XRD patterns of the two organic retarders used in this study are presented in Figure 3, which are (a) citric acid (ICDD PDF no. 00-015-0985) and (b) zinc acetate (ICDD PDF no. 00-033-1464).

Table 3 summarizes the oxide composition of the acrylic redispersible polymer powder from the XRF tests, as well as the elemental composition of the polymer powder from the EA analyses. The EA results suggest that about 71.1% of the polymer powder is composed of elemental carbon.

### 3.2. Compressive Strength

Regarding the initial setting of the mortars in Table 1, the mortar without retarders (Mixture M-0-N) quickly hardened within about 20 min of curing. However, the other mortars with retarders spent more curing time (at least 1 h) to harden, which would effectively improve the workability before placing it in actual construction.

**Figure 4:**
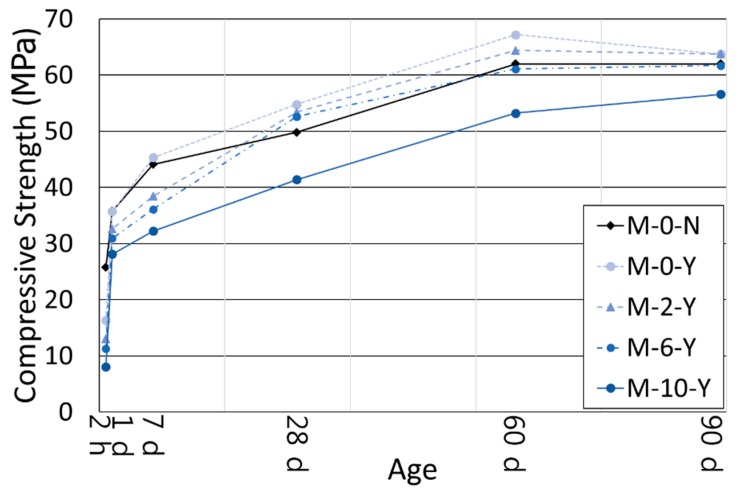
The compressive strengths of the mortars.

Figure 4 and Table 4 compare the compressive strengths of all mortar cases in Table 1, for curing ages of 2 h to 90 days. Of the cases with no polymer, the strength of the mortar without retarders (M-0-N) was approximately 25.8 MPa at 2 h of curing, which is about 41.6% of the strength at 90 days. In contrast, the mortar with retarders (M-0-Y) at 2 h showed a 36.8% lower strength than M-0-N. This confirms a typical delay in the strength development at the early age due to the retarders. However, the strengths of M-0-Y and M-0-N became similar at 1 day of curing, and furthermore, the former exhibited a higher strength than the latter after 7 days. The strength ratio between M-0-Y and M-0-N was the largest at 28 days of curing (approximately 1.10). The superior strength of M-0-Y to M-0-N at the long-term age was likely due to the effects of the retarders that increased the dispersibility of the cement grains, their specific surface areas, and their accessibility of water, which resulted in a finer, more ordered, and denser hydrated cementitious matrix [39,40,41].

Among the cases with retarders, an increase in the polymer amount induced a reduction in the mortar compressive strength at all the curing ages (Figure 4). This implies that the use of a higher polymer ratio caused a longer delay in the hydration of the cement blend in the presence of the retarders. Especially at 2 h of curing, the mortar with 10% polymer ratio (M-10-Y) showed a 50.9% strength reduction compared to the mortar with no polymer (M-0-Y). Moreover, the delaying effect of the polymer added to that of the retarders at the very early age, so that the strength of M-10-Y at 2 h was only approximately 8.0 MPa. Companion samples with no retarders [11] and several previous studies [42,43] also observed the delayed setting and strength development due to the use of redispersible polymer powders. It is supposed that this phenomenon was primarily attributed to entrained air by surfactants contained in the polymer powder, retention of water by micelles in the polymer emulsion, and partial or complete covering of cement hydrates by polymer films [42,43]. As the curing progressed, the strength ratio between M-10-Y and M-0-Y increased from 0.49 at 2 h to 0.89 at 90 days.

Even though the addition of the polymer caused a strength reduction in the mortars with retarders, those with a polymer ratio not more than 6% had a strength higher than or similar to Mixture M-0-N since 28 days of curing (Figure 4). This emphasizes the aforesaid effect of the retarders that caused the increased strength of the mortar at the long-term age.

The strength increase in the mortars became much slower after 1 day of curing, and there were only small changes of strength after 60 days of curing (Figure 4). For the five mortar mixtures, the strength ratio of 1 and 90 days ranged between approximately 49.6% and 57.7%, which are much higher than those of typical Portland cement concretes. At 90 days of curing, the compressive strengths of the four mortars excluding M-10-Y converged to approximately 62–64 MPa (Figure 4). Additionally, the strength of M-10-Y is expected to be comparable with the others at the long-term age exceeding 90 days; M-10-Y displayed a slow but steady growth of strength from 60 to 90 days (Figure 4). The strength convergence of the mixtures with different polymer ratios at the long-term age was likely attributed to the formation of a more refined load-carrying pore structure by the use of a higher polymer ratio, regardless of the inherent inferior strength of polymer powder [42]. More detailed discussions of the effects of the polymer on the hydration and microstructure of cement blend systems can be found in the companion paper [11].

### 3.3. Porosity

**Figure 5:**
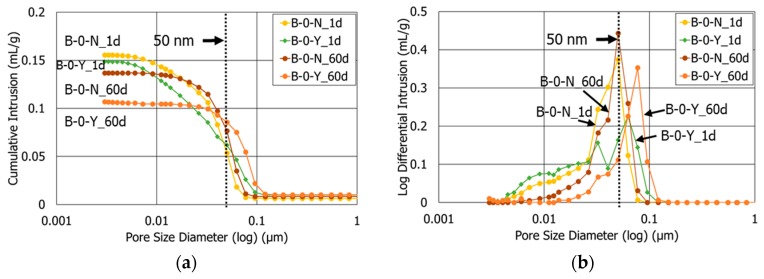
The pore size distributions of mixtures B-0-N and B-0-Y at different ages (1 d, 60 d): (**a**) cumulative intrusion; (**b**) log differential intrusion (unit: mL/g).

Figure 5 compares the pore size distributions of the HCP samples with no polymer (Mixtures B-0-N and B-0-Y) at 1 and 60 days of curing, acquired from the MIP tests. The test results of B-0-N (without retarders) and B-0-Y (with retarders) suggest that the total porosity and average pore diameter are considerably dependent on both the retarders and curing age (Table 5). With regard to the effect of the retarders, the total porosities of B-0-Y at 1 and 60 days of curing were 2.0% and 3.8% lower than those of B-0-N, respectively (Table 5). This is likely because the retarders increased the dispersion and specific surface area of anhydrous cement grains, which facilitated a finer and more ordered hydrated cementitious matrix after the first day of curing [40,41]. The lower porosity of B-0-Y is in accordance with the higher strength of M-0-Y compared with M-0-N after 1 day of curing (Figure 4). As for the effect of the curing age, the total porosity decreased in both mixture B-0-N and mixture B-0-Y as the curing progressed.

In contrast, the average pore diameters of both B-0-N and B-0-Y significantly increased at the curing age of 60 days from 1 day. It is noted that the volume of pores bigger than 50 nm in diameter (that is, macropores) increased as the curing progressed from 1 day to 60 days, whereas the volume of pores smaller than 50 nm in diameter (that is, micropores) decreased (Figure 5). This might be possible because the cement pastes without a polymer suffered considerable drying shrinkage during the air-curing process, compared with those containing the polymer powder, as observed in the authors’ preliminary study [9,10], which might cause the coalescences of micropores into macropores [44].

**Figure 6:**
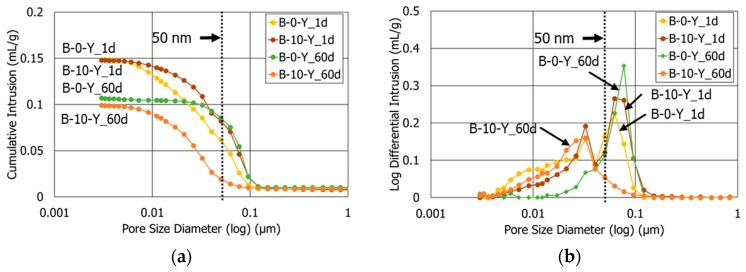
The pore size distributions of mixtures B-0-Y and B-10-Y at different ages (1 d, 60 d): (**a**) cumulative intrusion; (**b**) log differential intrusion (unit: mL/g).

Figure 6 compares the pore size distributions of the HCP samples with the retarders and different polymer amounts (Mixtures B-0-Y and B-10-Y) at the curing ages of 1 and 60 days. In general, the sample with a higher polymer ratio had a smaller porosity at both 1 and 60 days (Figure 6a and Table 5). The sample with 10% polymer ratio (B-10-Y) at 60 days of curing exhibited the least porosity among all the samples in this study, which signifies that the retarders and polymer together had synergistic effects in refining the pore structures of the cement pastes. In the authors’ companion study, the sample with only 10% polymer and no retarders showed a total porosity of 21.9% and an average pore diameter of 32.6 nm at 60 days of curing [11].

Contrary to the samples without polymer (B-0-N and B-0-Y), B-10-Y showed a continuous decrease in the average pore diameter from 1 to 60 days of curing (Table 5). A majority of pores of B-10-Y were macropores at 1 day of curing (Figure 6), indicating a slower strength development as discussed in Section 3.2. This is likely because the surfactants of the polymer powder entrained more air bubbles and amplified the average pore size at the early age [42,43]. After 60 days of curing, however, the vast majority of pores in B-10-Y were micropores (Figure 6).

### 3.4. Hydration Phase Evolution

**Figure 7:**
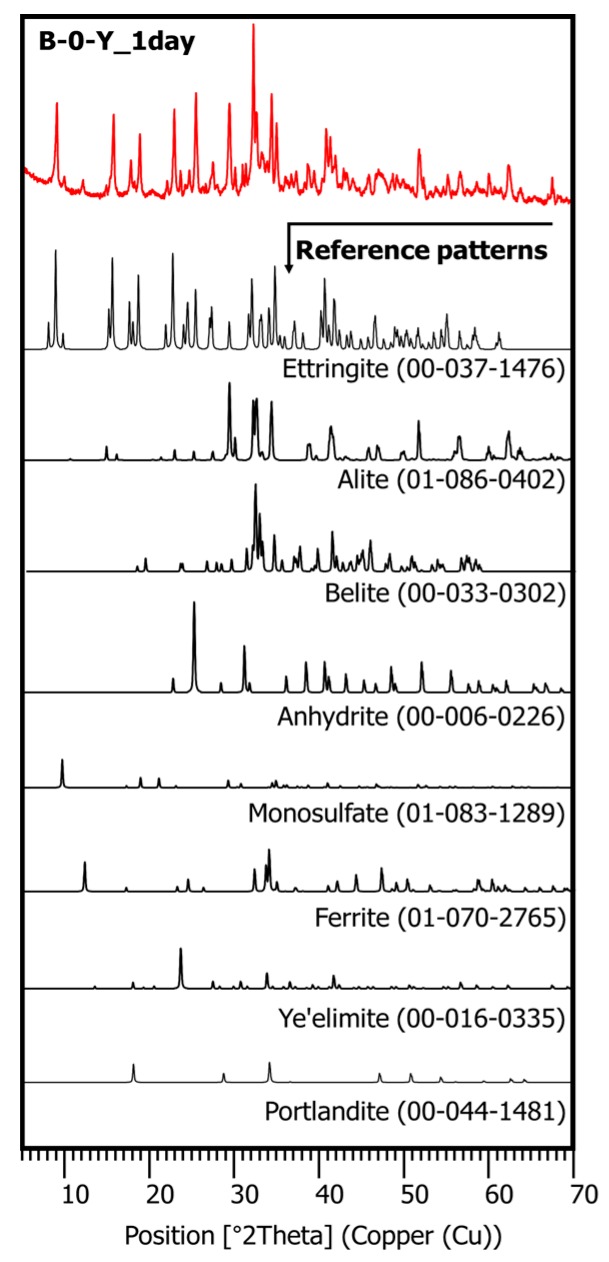
The integrated XRD pattern for mixture B-0-Y at 1 day.

**Figure 8:**
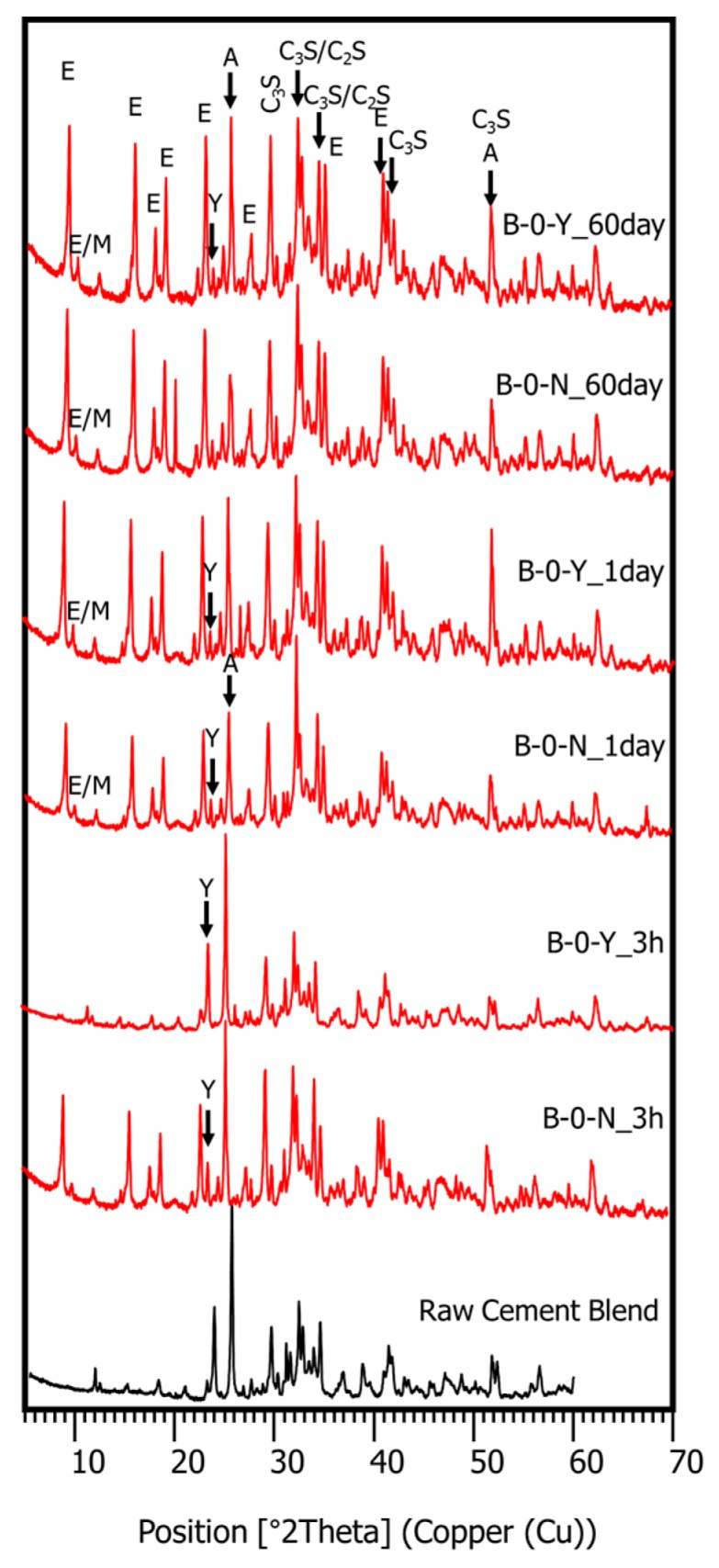
The XRD patterns of the cement pastes without a polymer (B-0-N, B-0-Y) at 3 h, 1 day, and 60 days.

**Figure 9:**
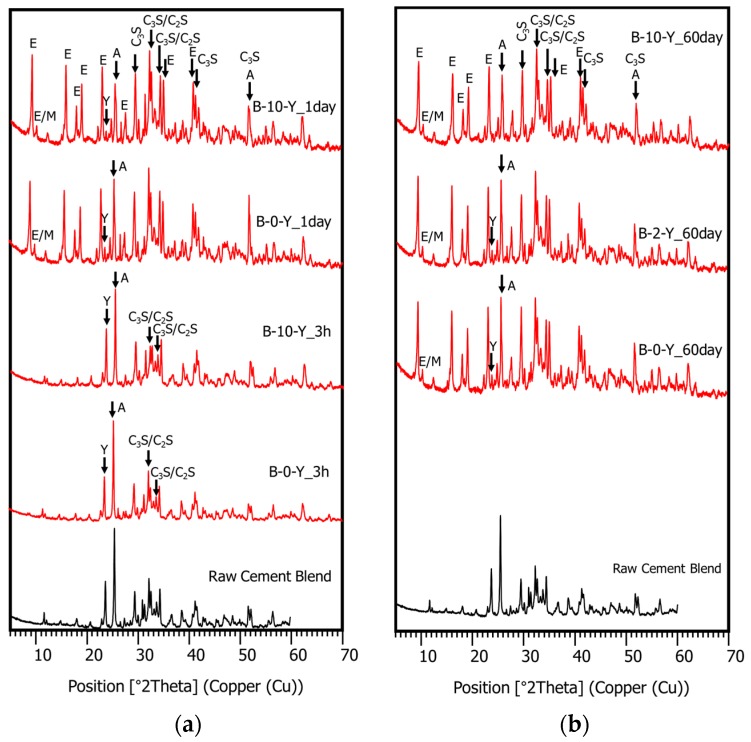
The XRD patterns of the cement blend pastes with varied polymer ratios: (**a**) B-0-Y and B-10-Y at 3 h and 1 day; (**b**) B-0-Y, B-2-Y and B-10-Y at 60 days.

**Figure 10:**
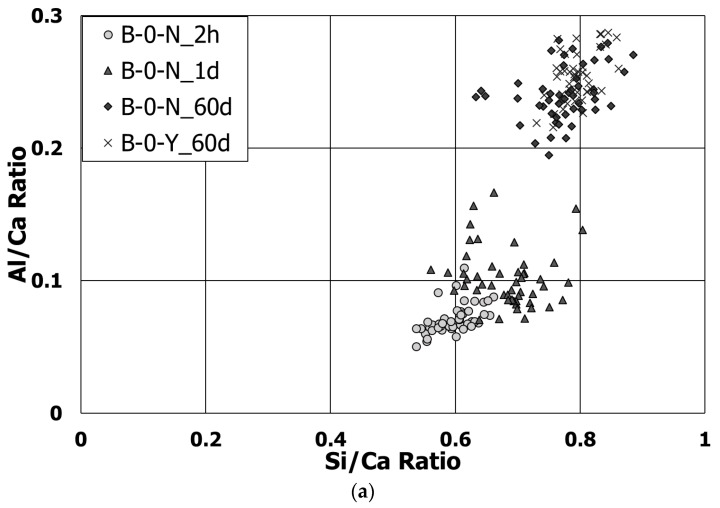

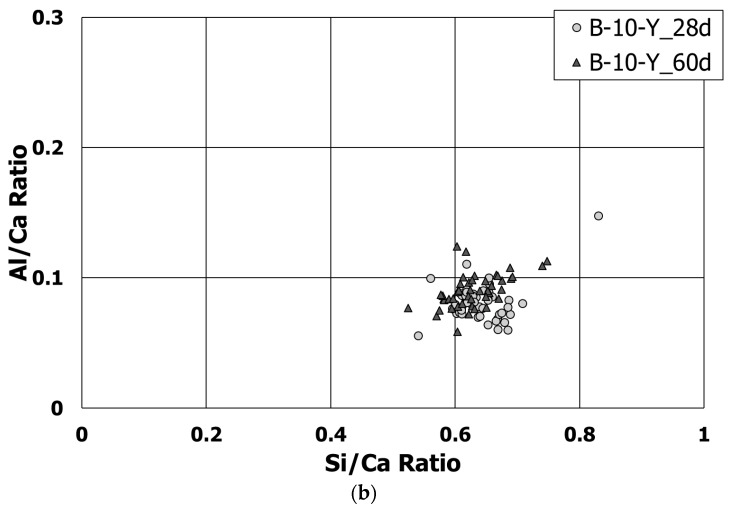
The Al/Ca vs. Si/Ca ratios for Mixtures (**a**) B-0-N and B-0-Y; and (**b**) B-10-Y at different ages.

XRD tests were conducted to investigate the effects of the retarders on the hydration process and the products of the cement pastes at different curing ages, with or without the polymer. In Figure 7, the XRD pattern of mixture B-0-Y at 1 day of curing displays the reflections of both hydrated and unhydrated mineral compounds. These include ettringite (ICDD PDF no. 00-037-1476), alite (ICDD PDF no. 01-086-0402), belite (ICDD PDF no. 00-033-0302), anhydrite (ICDD PDF no. 00-006-0226), monosulfate (ICDD PDF. no. 01-083-1289), ferrite (ICDD PDF no. 01-070-2765), ye’elimite (ICDD PDF no. 00-016-0335), and portlandite (ICDD PDF no. 00-044-1481). Although all the different cement pastes developed similar types of hydrated mineral phases, their proportions at a given curing age were significantly influenced by the presence of the retarders (Figure 8, Figure 9 and Figure 10).

Figure 8 presents the effects of the retarders on the XRD patterns of the cement pastes without a polymer (B-0-N vs. B-0-Y) at curing ages of 3 h, 1 day, and 60 days. In Figure 8 and Figure 9, “A” stands for anhydrite, “E” for ettringite, “A” for anhydrite, “M” for monosulfate, “Y” for ye’elimite, “C_3_S” for tricalcium silicate (alite), and “C_2_S” for dicalcium silicate (belite) (note that the *y*-axis values of the XRD patterns are square-rooted, and they are scaled such that the highest peak has the same height in all the different diffractograms). At 3 h of curing, the use of the retarders significantly inhibited the growth of ettringite; B-0-Y showed very small peaks of ettringite with high peaks of both ye’elimite (restrained by citric acid) and anhydrite. This is in accordance with the delayed strength development of M-0-Y at the early age of 3 h. At day 1 of curing, B-0-Y showed a significant growth of ettringite compared with B-0-N, which agrees with the strength test results that M-0-Y attained a similar strength to M-0-N at day 1, and became stronger than M-0-N after 7 days (Figure 4). At 60 days of curing, mixtures B-0-N and B-0-Y presented quite similar XRD patterns, which also accords with the similar strength results.

In the HCP samples without a polymer, monosulfate was barely detected at 3 h of curing, but it became apparent at 1 day of curing (Figure 8), which was possibly made from calcium sulfate (such as gypsum). The growth of monosulfate peaks in B-0-Y was more obvious than that in B-0-N. Winnefeld and Lothenbach [1] reported that monosulfate started to form after about 1–2 days of curing in their CSA cement-blended pastes when the formation of ettringite became less active. From day 1 to day 60, there were little changes of monosulfate in the HCP samples (Figure 8).

Figure 9a,b present the effects of the polymer on the XRD patterns of the cement pastes in the presence of the retarders. In general, the effects of the polymer were similar to those in the mixtures with no retarders, which are discussed in the companion paper [11]. The XRD pattern of B-10-Y at 3 h barely reflected the ettringite phase with high peaks of ye’elimite and anhydrite (Figure 9a). This was due to the combined effects of the retarders and polymer that additively delayed the hydration of the cement blend system at the very early age. At 1 day of curing, both B-0-Y and B-10-Y exhibited a rapid growth of ettringite peaks (Figure 9a). Meanwhile, regardless of the polymer ratio, the ye’elimite phase was markedly consumed within the first day of curing. Furthermore, at 60 days of curing, there were no noticeable differences in the XRD patterns of the samples with different polymer ratios (Figure 9b). This appears to reflect that the strengths of the mortar samples with different polymer ratios showed convergence at the long-term stage even in the presence of the retarders (Figure 4).

### 3.5. Morphological Transition

The SEM tests were conducted to identify the effects of the retarders on the morphological transitions of the polymer-modified cement blend systems at different curing ages. The compositions of hydrated or unhydrated products were measured by the EDS analyses at selected locations. For more detailed understanding on the hydration of the cement blend systems, the relationship between the Al/Ca and Si/Ca ratios, measured in a randomly selected and representative zone of 10 × 10 μm^2^ by the EDS spot analyses, was examined for the C-S-Hs of each sample (Figure 10 and Table 6). As the curing progressed, mixture B-0-N showed a clear trend of more Al uptake in the C-S-Hs with the increasing Si/Ca ratio. At 60 days of curing, B-0-Y had a slightly more Al uptake on average than B-0-N. This is likely to suggest that the samples with the retarders underwent a similar or slightly more progression of hydration at the long-term age. However, mixture B-10-Y exhibited little change of the Ca/Si ratio from 28 to 60 days of curing (Figure 10b), with a lesser Al uptake than mixture B-0-Y at a given curing age. This implies that, regardless of the presence of the retarders, the use of a higher polymer ratio led to a lower Al uptake of C-S-Hs of the cement blend systems; refer to the companion paper [11] for the cases with no retarders.

**Figure 11:**
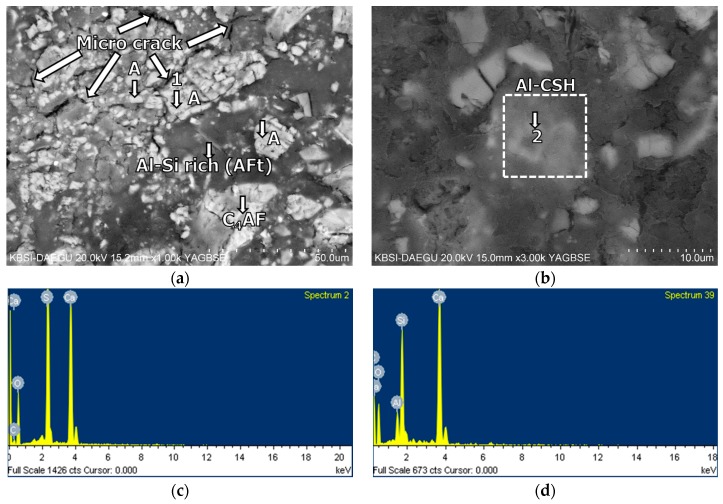
The microstructures of mixture B-0-N at 2 h: BSE images magnified by (**a**) ×1000 and (**b**) ×3000; EDS spectra of (**c**) point 1 in (**a**); and (**d**) point 2 in (**b**).

**Figure 12:**
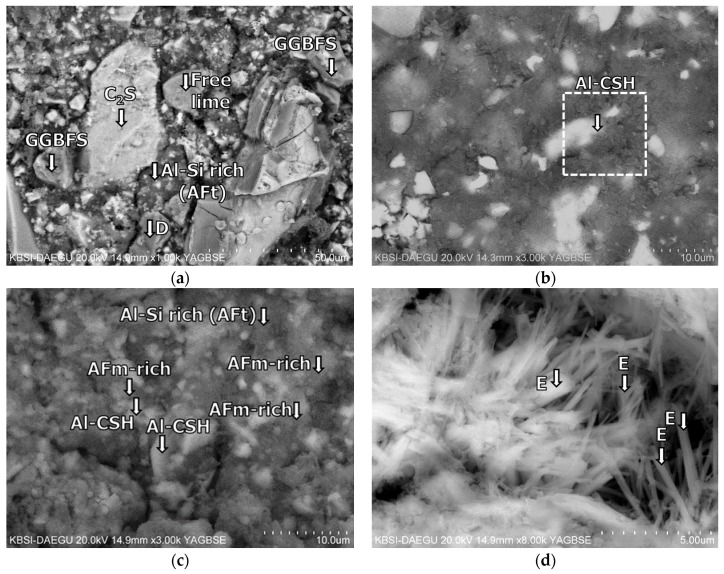
The microstructures of mixture B-0-N at 1 day: BSE images magnified by (**a**) ×1000; (**b**) ×3000; (**c**) ×3000; and (**d**) ×8000.

**Figure 13:**
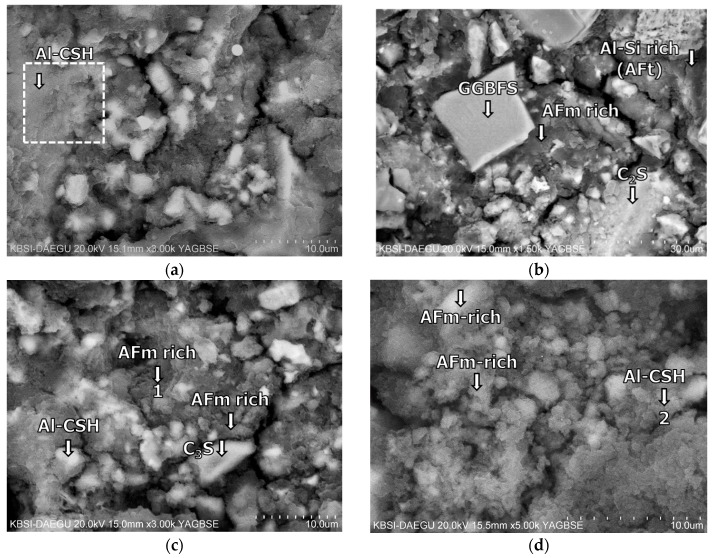

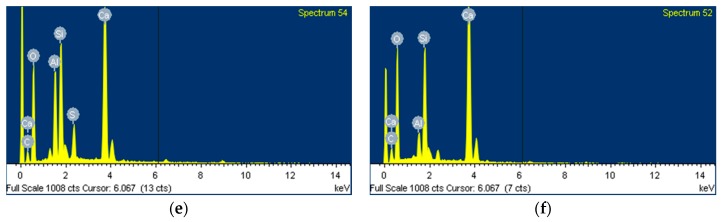
The microstructures of mixture B-0-N at 60 days: BSE images magnified by (**a**) ×3000; (**b**) ×1500; (**c**) ×3000 and (**d**) ×5000; EDS spectra of (**e**) point 1 in (**c**); and (**f**) point 2 in (**d**).

**Figure 14:**
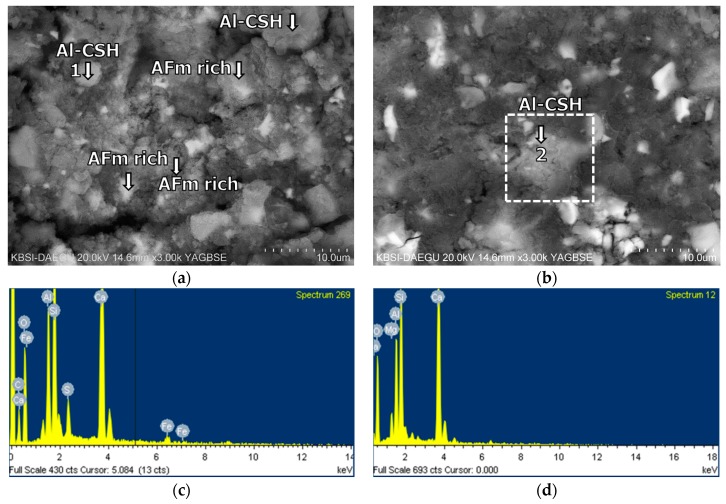
The microstructures of mixture B-0-Y at 60 days: BSE images magnified by (**a**) ×3000; and (**b**) ×3000; EDS spectra of (**c**) point 1 in (**a**); and (**d**) point 2 in (**b**).

**Figure 15:**
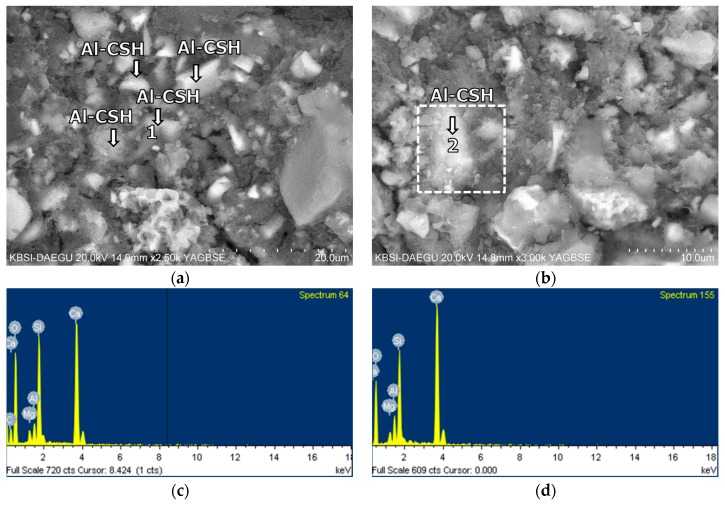
The microstructures of mixture B-10-Y at 28 days: BSE images magnified by (**a**) ×2500; and (**b**) ×3000; EDS spectrum of (**c**) point 1 in (**a**); and (**d**) point 2 in (**b**).

**Figure 16:**
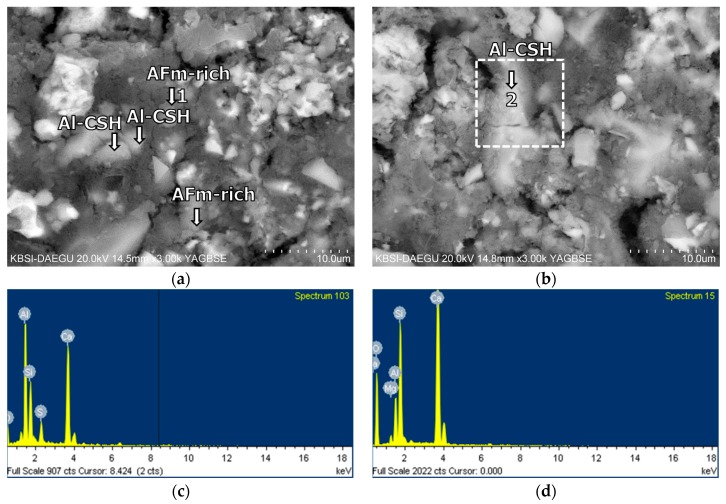
The microstructures of mixture B-10-Y at 60 days: BSE images magnified by (**a**) ×3000; and (**b**) ×3000; EDS spectra of (**c**) point 1 in (**a**); and (**d**) point 2 in (**b**).

The BSE images of mixture B-0-N at 2 h of curing are given in Figure 11. In Figure 11, Figure 12, Figure 13, Figure 14, Figure 15 and Figure 16, “A” stands for anhydrite, “C_4_AF” for tetracalcium aluminoferrite, “C_3_S” for tricalcium silicate, “D” for dolomite, “C_2_S” for dicalcium silicate, and “E” for ettringite. A substantial amount of unhydrated phases such as cement clinker, anhydrite, C_2_S, C_3_S, and C_4_AF were identified at 2 h. Additionally, there were significant footprints of hydrated phases in complex forms (indicated as “Al-Si rich” in Figure 11) containing both the ettringite (AFt) and amorphous alumina phases. After 1 day of curing, B-0-N exhibited a fast growth of ettringite (Figure 12). In addition, the monosulfate phases (indicated as “AFm-rich” in Figure 12), which were not seen at 2 h of curing, but were visible after 1 day of curing. After 60 days of curing (Figure 13), there were much smaller unhydrated phases than the early ages, as identified by the XRD tests (Figure 8).

From the early age of 2 h, mixture B-0-N also developed Al-rich calcium silicate hydrates (C-S-Hs); the average calcium-to-silica (Ca/Si) ratio was 1.68, 1.47, and 1.30 at 2 h, 1 day, and 60 days, respectively (Table 6). For reference, the Ca/Si molar ratio of C-S-Hs in Portland cement pastes ranges between 1.2 and 2.3 after 1 day to 3.5 years [45,46]; the ratio is primarily governed by the type of binder, water/binder ratio, and curing age. At 60 days of curing, the average Ca/Si ratios of C-S-Hs in B-0-N and B-0-Y were very similar, which were 1.30 and 1.26, respectively (Table 6). These similar Ca/Si ratios seem to reflect similar degrees of hydration, which was evident from the convergent strengths of M-0-N and M-0-Y at the long-term age.

Mixture B-10-Y at 28 days of curing also contained both the unhydrated and hydrated phases (Figure 15). B-10-Y at 60 days of curing showed no significant change of morphology from 28 days, showing the same types of unhydrated and hydrated phases (Figure 16). At 60 days, the Al-rich C-S-Hs of B-10-Y had a higher average Ca/Si ratio of 1.59 than those of B-0-N and B-0-Y (that is, 1.30 and 1.26). This supports that the hydration progress of B-10-Y was hindered by the presence of the polymer. This finding also substantiates the lowest strength development of Mixture M-10-Y at all curing ages (Figure 4).

## 4. Conclusions

In this study, five mixtures of cement pastes and five mixtures of mortars were tested to investigate the effects of set retarders on the hydration, microstructure, and strength development of polymer-modified CSA blended cement systems at curing ages from 2 h to 90 days. Overall, the use of retarders proved to be an effective method for improving workability for the fast-setting cement blend systems without sacrificing and rather even enhancing the ultimate strength. The findings and conclusions of this study are summarized as follows:

1. The XRD and SEM results show that the growth of the hydrated phases (for example, ettringite, monosulfate, C-S-Hs) was substantially restrained with the retarders at the very early age (that is, 2–3 h). This likely occurred because the retarders formed hydrophobic barrier layers surrounding anhydrous mineral phases (for example, anhydrite, ye’elimite, C_3_S, C_2_S, C_3_A) in the finely dispersed cement grains. Moreover, the delaying effect of the retarders cumulatively added to the delaying effect of the polymer.
2. The use of the retarders increased the ultimate strength of the cement blend systems at the long-term age. Even with a polymer ratio up to 6%, the mortars with the retarders showed higher compressive strengths than the mortar without both retarders and polymers after 28 days of curing. This was likely to happen because the retarders created a finer and denser hydrated cementitious matrix as observed in the MIP results, by increasing the dispersibility of cement grains, their specific surface areas, and the accessibility of water to them.
3. Despite variations in the polymer ratio, the compressive strengths of all the mortars with retarders tended to converge at the age of 90 days. This reflects the formation of a more refined pore structure with a higher polymer ratio that compensated the inherited weak strength of the polymer powder itself, as well as the formation of a monolithic co-matrix between the cement hydrates and polymer phases. The authors will examine the combined effects of the set retarders and polymer powder on the ITZs in the near future.
4. At the age after 60 days, the sample with retarders and 10% polymer exhibited both the smallest porosity and average pore diameter among all the HCP samples. This highlights that the combined use of retarders and polymer had a synergetic effect to refine the pore structures of the cement blend systems.
5. According to the EDS spot analyses, the paste with the retarders at 60 days of curing had a slightly more Al uptake on average than the paste with no retarders. This supports the compression test results that the mortar with the retarders showed higher strengths than the mortar without retarders after 28 days of curing.

## Figures and Tables

**Table 1:** The mix proportions of the tested mortars and cement pastes.

**Mixture Label**	**Mortar (Unit: kg/m^3^)**									
	**Water**	**Cement**		**Sand**		**Polymer**		**Retarder A**		**Retarder B**
M-0-N	339	892		892		0		0		0
M-0-Y	339	892		892		0		1.43		1.07
M-2-Y	339	892		892		17.9		1.43		1.07
M-6-Y	339	892		892		53.6		1.43		1.07
M-10-Y	339	892		892		89.3		1.43		1.07
**Mixture Label**	**Cement Paste (Unit: kg/m^3^)**									
	**Water**		**Cement**		**Polymer**		**Retarder A**		**Retarder B**	
B-0-N	512		1346		0		0		0	
B-0-Y	512		1346		0		2.15		1.62	
B-2-Y	512		1346		26.9		2.15		1.62	
B-6-Y	512		1346		80.8		2.15		1.62	
B-10-Y	512		1346		134.6		2.15		1.62	

**Table 2:** The chemical oxide composition of the raw cement blend from X-ray fluorescence.

Oxide in Cement Blend (wt.%)						
CaO	SO_3_	SiO_2_	Al_2_O_3_	MgO	Fe_2_O_3_	Others
54.6	14.6	12.4	11.8	2.3	2.3	2.0

**Table 3:** The XRF and elemental analysis results of the polymer powder (unit: wt.%).

**Oxide Composition (wt.%)**	**CaO**	**SO_3_**	**SiO_2_**	**Others**
	24.9	45.9	26.2	3.0
**Element Composition (wt.%)**	**Carbon**	**Hydrogen**	**Sulfur**	**Nitrogen**
	71.1	7.7	5.3	0.7

**Table 4:** The compressive strengths of the mortars (unit: MPa).

Age	2 h	1 Day	7 Days	28 Days	60 Days	90 Days
Mixture Label	Compressive Strength (MPa)					
	Average (std. dev.)	Average (std. dev.)	Average (std. dev.)	Average (std. dev.)	Average (std. dev.)	Average (std. dev.)
M-0-N	25.8 (0.14)	35.8 (2.65)	44.1 (1.72)	49.8 (4.21)	62.0 (3.84)	62.0 (4.17)
M-0-Y	16.3 (0.60)	35.7 (1.05)	45.3 (2.20)	54.8 (5.91)	67.2 (4.34)	63.7 (0.75)
M-2-Y	13.0 (0.07)	32.6 (1.38)	38.4 (0.90)	53.4 (2.40)	64.4 (4.92)	63.8 (5.65)
M-6-Y	11.3 (0.35)	30.9 (1.40)	36.1 (1.54)	52.6 (2.14)	61.1 (5.39)	61.7 (1.94)
M-10-Y	8.0 (0.08)	28.1 (1.00)	32.2 (0.15)	41.4 (1.64)	53.2 (2.18)	56.6 (4.26)

**Table 5:** The total porosity and average pore diameter of each mixture.

Mixture Label	Age (day)	Total Porosity (%)	Average Pore Diameter (nm)
B-0-N	1	27.5	27.9
	60	23.8	42.8
B-0-Y	1	25.5	23.6
	60	20.0	52.4
B-10-Y	1	24.5	34.5
	28	20.1	21.0
	60	18.1	21.4

**Table 6:** The average Ca/Si and Al/Ca ratios of Al-rich C-S-Hs of each mixture.

Mixture Label	Curing Age	Average Ca/Si Ratio	Average Al/Ca Ratio
B-0-N	2 h	1.68	0.072
	1 day	1.47	0.103
	60 days	1.30	0.242
B-0-Y	60 days	1.26	0.254
B-10-Y	28 days	1.57	0.082
	60 days	1.59	0.091
